# Investigation of Acupuncture Sensation Patterns under Sensory Deprivation Using a Geographic Information System

**DOI:** 10.1155/2012/591304

**Published:** 2012-11-21

**Authors:** Florian Beissner, Irene Marzolff

**Affiliations:** ^1^Pain & Autonomics-Integrative Research (PAIR), Department of Psychiatry and Psychotherapy, Jena University Hospital, 07743 Jena, Germany; ^2^Institute for Physical Geography, Goethe University, 60438 Frankfurt am Main, Germany

## Abstract

The study of acupuncture-related sensations, like *deqi* and propagated sensations along channels (PSCs), has a long tradition in acupuncture basic research. The phenomenon itself, however, remains poorly understood. To study the connection between PSC and classical meridians, we applied a geographic information system (GIS) to analyze sketches of acupuncture sensations from healthy volunteers after laser acupuncture. As PSC can be subtle, we aimed at reducing the confounding impact of external stimuli by carrying out the experiment in a floatation tank under restricted environmental stimulation. 82.4% of the subjects experienced PSC, that is, they had line-like or 2-dimensional sensations, although there were some doubts that these were related to the laser stimulation. Line-like sensations on the same limb were averaged to calculate sensation mean courses, which were then compared to classical meridians by measuring the mean distance between the two. Distances ranged from 0.83 cm in the case of the heart (HT) and spleen (SP) meridian to 6.27 cm in the case of the kidney (KI) meridian. Furthermore, PSC was observed to “jump” between adjacent meridians. In summary, GIS has proven to be a valuable tool to study PSC, and our results suggest a close connection between PSC and classical meridians.

## 1. Introduction

Acupuncture is a medical intervention originating from ancient Asia, where needles are used to stimulate certain points on the body. Despite more than five decades of intensive research in Asia and the West, the underlying mechanisms of acupuncture are still largely unknown. A phenomenon that may be of great importance for the understanding of these mechanisms is a specific sensation upon stimulation of acupuncture points that is called *deqi* (*“the arrival of qi”, 得氣*) in Chinese medicine. Previous works have shown that the perception of this sensation usually described by words like “aching,” “soreness,” “pressure,” or “tingling” [1] is similar between subjects, irrespective of their expectation, sex, or cultural background [2–4]. Judging from the adjectives that are most frequently used to describe *deqi*, it is a mixed sensation with a strong component of C-fiber-mediated pain [5]. Another important feature of *deqi* is that it often spreads or radiates from the point of its elicitation. This has led to the term “propagated sensation along meridians” (PSM) or, more commonly, “propagated sensation along channels” (PSC) [2].

So far, the investigation of PSC has been limited by a lack of appropriate methods for its assessment. Questionnaires like the MASS (MGH acupuncture sensation scale) [6] have been developed to measure sensory qualities and intensity of *deqi*. But they give only a rough estimate of the spreading/radiation experienced by the subject. To gain a deeper understanding of PSC, however, it is crucial to measure its exact course and compare it between subjects.

To close this gap, we developed a new method by combining standardized subjects' drawings, a method often used for pain assessment [7], with an analysis based on a geographic information system (GIS) [8]. Geographical information systems allow to map, visualize, and analyze the patterns, dimensions, relationships, and changes of spatial data. The spatial reference is usually a coordinate system of the Earth such as latitudes and longitudes, metric units in a map projection or postal codes, but any other local system may be used as well. In health care and medical science, spatial data analyses on local to global scales have a long tradition, beginning with the classic study of London's 1854 cholera epidemic by British epidemiologist John Snow [9]. GIS today plays an important role in public and private health care, medical research and insurance for management, planning and analysis [10, 11], but few studies have so far been conducted on the basis of body maps. Our approach allows for the first time to map the detailed course of PSC in a sample of subjects, compare the results between subjects, and calculate mean courses. The results can then be compared to the so-called *meridians* (chin.: *“jingluo”*, *经络*), vessel-like structures that according to Chinese medical theory traverse the human body circulating an immaterial substance (chin.: *“qi”, 氣*). Based on acupuncture classics as well as previous reports, we hypothesized a strong resemblance between the courses of certain meridians and those of PSC. Since no anatomical correlate has been found for the concept of meridians despite decades of research [12, 13], it is very likely that these structures were originally inspired by the line-like appearance of PSC patterns.

As PSC can sometimes be subtle, we aimed at reducing the confounding impact of tactile stimuli that can hardly be controlled under normal circumstances. Therefore, all measurements were carried out in a floatation tank [14] under restricted environmental stimulation [15]. A floatation tank is filled with saline that is constantly kept at skin temperature as is the surrounding air. It is soundproof and either dark or illuminated by very dim light. The subject inside the tank floats supine on the saline without effort, which greatly reduces the amount of tactile, visual, and auditory input and allows full concentration on sensations inside the body.

## 2. Methods

### 2.1. Study Design

**Figure 1:**
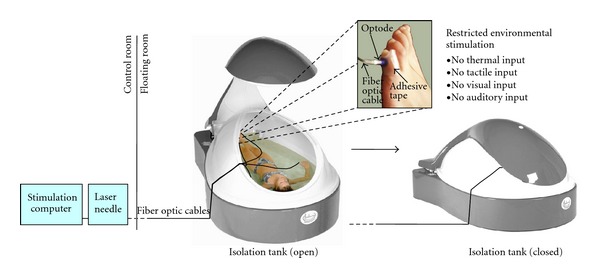
Experimental design. The subject inside the tank was stimulated with an acupuncture laser, while floating on the water surface to reduce tactile input. Laser light was transmitted by fiber optic cables from the control room. Laser optodes were attached to the skin using silicone adapters and adhesive tape. After the tank was closed, there was also reduced visual, auditory, and thermal input, so that subjects could concentrate on sensations occuring during laser acupuncture. Tank images courtesy of Floataway, Norfolk, UK.

All measurements were carried out at floatbase GmbH (Frankfurt am Main, Germany). The experimental design is shown in Figure 1. When subjects arrived at the floatbase, they were first familiarized with the isolation tank and its emergency facilities to reduce anxiety. Next, subjects were shown the questionnaire with the body schemes, which were used after the experiment to sketch their sensations. Silicone adapters for the laser optodes were attached to the following points of the body using adhesive tape: (1) Little toe of the left foot, acupuncture point Bladder 67 (BL-67), (2) Big toe of the right foot, acupuncture point Spleen 1 (SP-1), (3) Index finger of the left hand, acupuncture point Large Intestine 1 (LI-1), (4) Little finger of the right hand, acupuncture point Small Intestine 1 (SI-1). For the exact localization of the points, see Supplementary Figure 1 in Supplementary Material available online at doi:10.1155/2012/591304. Subjects were instructed how to enter the tank, take a comfortable position, insert the laser optodes into the silicone adapters, and close the tank from the inside. They were also informed about the timing of the experiment, which is described in detail below. The investigator then left the room so that subjects could undress and enter the tank.

The closing of the tank was indicated to the investigator by a signal light and marked the official beginning of the experiment. In a first period, lasting 10 minutes, subjects did not receive any stimulation, so they could adapt to the new environment. In the second period, lasting 16 minutes, a randomly chosen set of three of the four points were stimulated one after another with the laser in a randomized order. Each stimulation lasted three minutes followed by one minute without stimulation. For the point that was not stimulated, the laser was simply left switched off during the entire four (3 + 1) minutes. The end of the stimulation paradigm was indicated to the subjects by a special sound (sea rushing). From this point, subjects had four minutes to prepare themselves to leave the tank. After this time, the tank opened automatically.

After taking a shower to remove excess saline from the body, subjects were handed out the questionnaire, which they were asked to fill out immediately.

### 2.2. Subjects

20 healthy subjects (10 male/10 female) took part in the study. The mean age was 28.8 ± 4.1 (S.D.) years. Prior to the measurement, subjects were screened for any acute diseases or contraindications for using the floatation tank (skin diseases, epilepsy, claustrophobia, and pregnancy). All subjects were healthy on the day of the measurement. None of them had any history of neurological disease or took any kind of medication on a regular basis. The intake of analgesics as rescue medication was prohibited during the five days before the measurement. None of the subjects had any prior knowledge of acupuncture theory as a student or practitioner.

After the measurement, three subjects (1 male, 2 female) were excluded. Two mentioned the intake of antihistaminic drugs not until after the measurement, and one developed severe vertigo and nausea inside the tank.

### 2.3. Laser Stimulation Device

Low-level laser stimulation was administered using a Laserneedle (Laserneedle EG GmbH, Wehrden, Germany) emitting a combination of 655 nm (red) and 785 nm (infrared) laser light with an irradiation power of about 15 W/cm² at the distal output. Laser optodes were applied in contact mode using silicone adapters, which were attached to the skin using adhesive tape. The laser power at the distal output of each optode was about 40 mW. Reflection losses could be neglected due to the direct contact of the optode with the skin. Acupuncture Lasers like this have been used before in a number of studies [16, 17].

### 2.4. Isolation Tank

All measurements were performed using the same isolation tank (floataway, Norfolk, UK) at floatbase. The size of the tank was 2.20 m × 1.50 m with a water depth of 25 cm. The water inside the tank was loaded with 300 kg magnesium sulfate to achieve the floatation effect, that is, make subjects float on its surface. The water temperature was kept at a constant 34°C to minimize temperature sensations. The tank and the surrounding room were soundproof and subjects were asked to keep their eyes shut throughout the whole experiment, thus minimizing auditory and visual sensations. A dim red light illuminated the tank during the whole experiment to prevent subjects from noticing changes in ambient light due to switching of the laser.

### 2.5. Body Schemes and Questionnaires

To assess and compare bodily sensations experienced by the subjects, body schemes were developed based on the illustrations in [18] (see Supplementary Figure 1). When these body schemes were first shown to the subjects before the measurements, the following instructions were given: “Please pay attention to any sensation that you believe is an effect of the laser stimulation. You will later be asked to sketch your sensations on these body schemes. You will also be asked about the quality and intensity of the sensations.” Directly after the end of the measurements, subjects were given the body schemes as well as the German version of the McGill pain questionnaire [19, 20] with its 77 descriptors (sensory, affective, and evaluative) and an additional visual analogue scale. The order of the descriptors was randomized for each subject. The following instructions were read aloud to the subjects: “Please describe any sensation that you believe was an effect of the laser stimulation. Sketch the localization of these sensations on the body schemes using the following three signs: A dot for every point-like sensation, a line for every line-like sensation and hachures for every two-dimensional sensation. Please choose any number of descriptors from the questionnaire that describe the sensations you have experienced. Please rate the overall intensity of these sensations on the vertical line between 0 (no sensation) and 100 (maximally tolerable sensation). Finally, please indicate, which of the points you believe have been stimulated and, what your perceived order of stimulation was.”

### 2.6. Analysis of Psychophysical Data

Descriptors chosen by the subjects to describe their sensations were ranked by their absolute frequency (see Table 1). Only those descriptors that were chosen five times or more were taken into account.

Based on subjects' estimation concerning the point selection, that is, the three stimulated points out of four points with attached optodes, results were sorted into four standard categories for every point: hits, misses, correct rejects, and false positives (see Table 2). The sum over all subjects was calculated for each category. A one-tailed Fisher's exact test was used to analyze a possible connection between stimulation and perception. A *P* value of <0.05 was considered significant.

Finally, the perceived order of stimulation was compared to the actual order.

### 2.7. Analysis of the Sketched Sensations

In order to compare the localization and extent of the sensations between subjects, a GIS body-map template (front and back) was created in ESRI ArcGIS 10.0 by digitizing the body scheme used in the questionnaire and scaling it to the mean body height of all subjects (175 cm). All body sketches were scanned, transferred into the GIS database, and georeferenced to the body-map template. The database was then populated with the subjects' mapped sensations in point, polyline, and polygon format by digitizing them on-screen from the scanned sketches, and attributing each feature with a key ID and the subject code. For point-like sensations, the radius as drawn in the sketch was added to the attribute table and for line-like and two-dimensional sensations, the line lengths or polygon area, respectively, were calculated in the GIS (see Supplementary Table 1). In the case of multiple objects of the same kind for one subject (e.g., two line-like sensations), these objects were given different roman numbers as indices. Note that GIS calculations in this case were based on a 2D representation of the body, not taking account of the actual 3D surface of the body.

**Figure 2:**
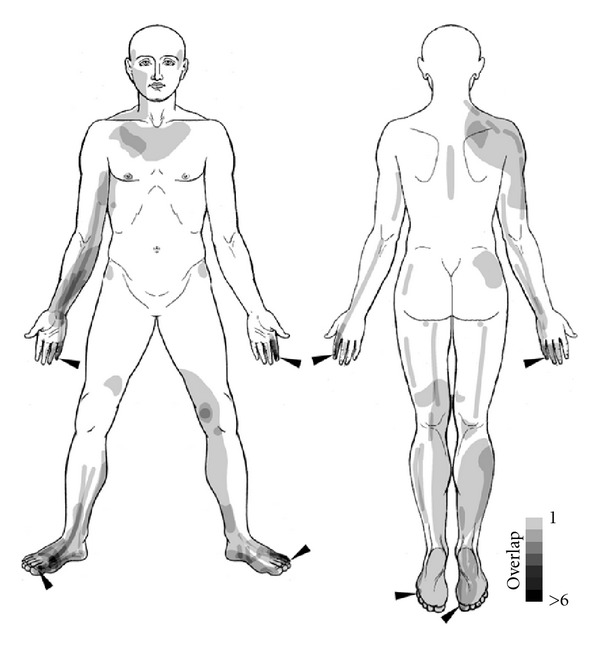
Exploratory GIS analysis of subjects' sensations experienced during laser acupuncture. The grey level of the superimposed polygons indicates the number of subjects who experienced a point-like, line-like or 2-dimensional sensation in this area.

To be able to overlay all sensations in one plot (see Figure 2), lines representing the line-like sensations were converted to polygons by a buffering algorithm in the GIS, resulting in 1.5 cm wide swaths. Point-like sensations were converted to circles using the radius drawn in the sketches by the subjects. All sensations were then overlaid and intersected, resulting in a dataset of overlapping sensation polygons. The choice of 1.5 cm width for the line-like sensations was based on previous literature reports on PSC [21].

### 2.8. Calculation of Sensation Mean Courses and Comparison to Classical Meridians

Depending on the subject's sensation and way of sketching, the line-like sensations were represented by single or multiple line features, sometimes arranged in parallel or radially. In order to analyze their general direction and compare them to classical meridians, mean courses of all lines related to a certain body part (e.g., lower right leg) were calculated as follows: each line was subdivided into 10 parts of equal length that were numbered 1 to 10 and attributed with their length. For all lines belonging to the same body part, the length-weighted mean center point of each group of sublines with the same part number was then calculated. Finally, the resulting 10 center points were converted back to a polyline, thus representing a mean course and length of the mapped sensations within a given body part. Only sensation mean courses longer than 5 cm were further considered.

**Figure 3:**
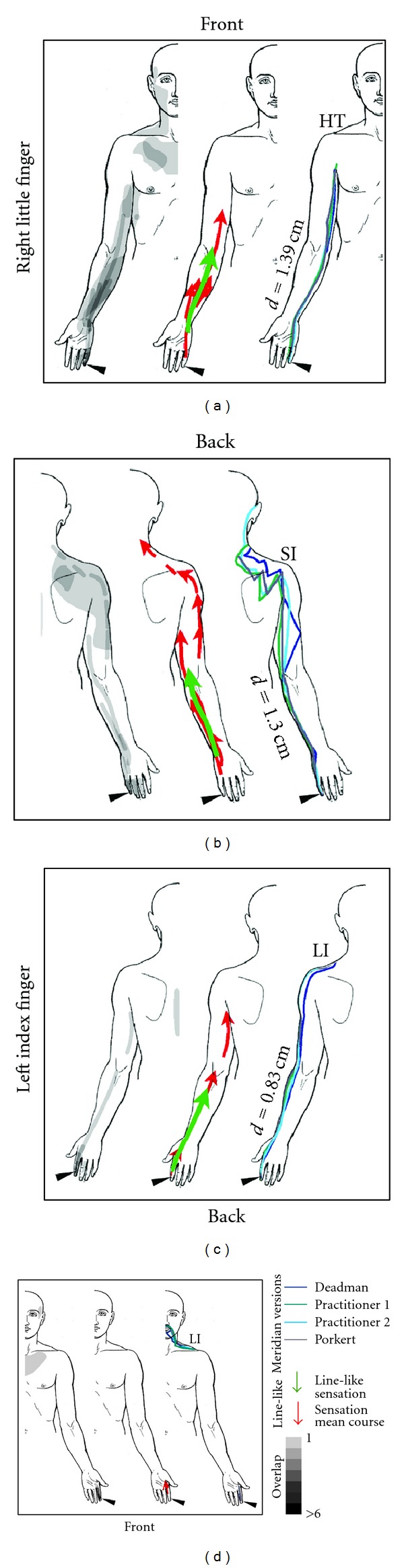
Comparison of subjects' sensations and the courses of meridians on the upper body. In each frame, the left image depicts all sensations in the respective quadrant of the body (see also Figure 2). The middle image shows all line-like sensations as well as sensation mean courses. The right image depicts the course of the meridian taking into account differences from the literature. The mean distance of the sensation mean course from the respective meridian as calculated with the GIS is given and denoted with a small d (for explanation see Supplementary Figure 2). For the minimum and maximum distance, the reader is referred to Table 3.

**Figure 4:**
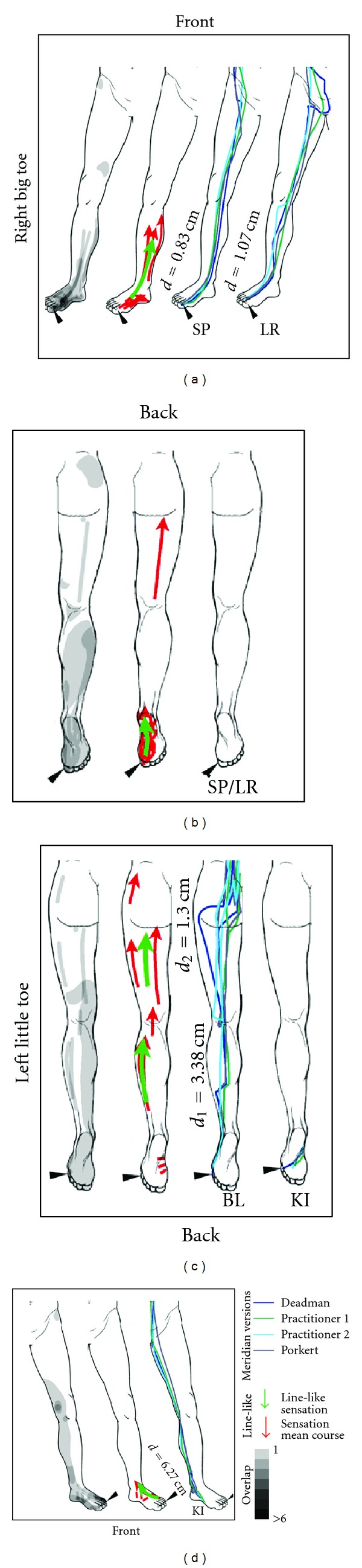
Comparison of subjects' sensations and the courses of meridians on the lower body. In each frame, the left image depicts all sensations in the respective quadrant of the body (see also Figure 2). The middle image shows all line-like sensations as well as sensation mean courses. The right image depicts the course of the meridian taking into account differences from the literature. The mean distance of the sensation mean course from the respective meridian as calculated with the GIS is given and denoted with a small *d* (for explanation see Supplementary Figure 2). For the minimum and maximum distance, the reader is referred to Table 3. Note, that for the right big toe, none of the relevant meridians (SP/LR) runs on the backside of the leg.

To be able to compare sensation mean courses to classical meridians, an approach was needed to incorporate the variability of different literature sources concerning the exact course of meridians. We decided to include two well-known reference works [22, 23] as well as drawings from two specialists, each with more than 10 years of experience in application and teaching of traditional chinese medicine. While the latter two drew their sketches directly on the body scheme, meridian courses from the reference works were transferred to the schemes by one of the authors (FB). After scanning and georeferencing the sketches, the four versions of all meridians were digitized and added to the GIS database. For each of the four versions, distance maps in the form of raster datasets with 1 cm resolution were computed by calculating the Euclidean distance for each raster cell to the meridian. The four versions were then averaged using Map Algebra, yielding a map of mean distance to each meridian (see Supplementary Figure 2). Using a GIS tool originally developed to extract 3D properties such as mean elevations for features located on a terrain surface, the minimum, maximum, and mean distance for each sensation mean course recorded by the subjects could then be calculated (Table 3, Figures 3 and 4).

## 3. Results

### 3.1. Psychophysical Data

The mean intensity of subjects' sensations had a VAS score of 26.47 ± 20.09 (SD) (see Supplementary Table 2).

Subjects chose 7.76 ± 5.23 (mean ± SD) descriptors for their sensations. Eleven descriptors were chosen by five or more subjects (see Table 1). These were *tingling, radiating, spreading, hot, dull, pulsing, throbbing, pricking, stinging, tender, *and *pinching*. It should be mentioned that some subjects, while filling out the questionnaire, mentioned the lack of the descriptor *warm* that seemed to describe their sensations. As no further explanations by the experimenter were allowed, most of them decided to use the descriptor *hot *instead.

Five subjects correctly identified the three out of four points that had been stimulated (see Table 2). Nine subjects missed one or more points, and seven subjects had false positives, that is, one of the points they chose had not been stimulated. Fisher's exact test over all points (pooled data) was significant (*P* < 0.02) showing a connection between stimulation and perception. For the single points, however, only LI-1 at the left index finger showed a significant result (*P* < 0.03). 

Interestingly, the order of stimulation of the points was not estimated correctly by a single subject.

### 3.2. Sketched Sensations

13 subjects experienced point-like, 12 line-like, and 13 2-dimensional sensations during laser acupuncture. 10 subjects experienced all three kinds of sensations. The detailed results of the GIS analysis can be found in Supplementary Table 1. While the majority of subjects (10) reported all three kinds of sensations, two subjects drew only points, and one returned an empty body scheme. Excluding the latter three subjects, we can, thus, say that 14 out of 17 subjects (82.4%) experienced PSC. The mean radius of the point-like sensations was 1.67 ± 1.35 cm. Line-like sensations had a mean length of 13.81 ± 11.81 cm, while the average length per subject of all summed up single lines was 38.19 ± 40.50 cm. 2-dimensional sensations had a mean area of 67.31 ± 94.72 cm^2^  or 201.94 ± 222.87 cm^2^ , if summed up per subject. Figure 2 shows an overlay of all sensations with dots represented by circles and lines represented by swaths. The majority of sensations were reported from the limbs, while relatively few subjects sketched sensations on the trunk. Point-like sensations were mostly restricted to the stimulation loci. One eye-catching exception was a point experienced by one subject bilaterally at the location of acupuncture point BL-36, directly below the buttock. Furthermore, for the SI Meridian, there was a single subject showing line-like sensations almost along the whole course (see lines in the back shoulder region in the upper right of Figure 3).

### 3.3. Comparison of Sensation Mean Courses to Classical Meridians

There were eight subregions on the limbs were sensation mean courses could be calculated. These are shown in Figure 3 (for the upper half of the body) and Figure 4 (for the lower half of the body). Three of the mean courses were on the front (inner side of the right arm, inner and outer side of the left arm), and five lines on the back side of the body scheme (inner side of the right leg, sole of the right foot, dorsum of the left foot, and posterior thigh area). The mean length of the sensation mean courses was 12.33 ± 7.32 cm.

The assessment of distances between sensation mean courses and classical meridians (see Table 3) showed an overall good agreement between the two. The smallest mean deviation of the sensation mean from the meridian course was 0.83 cm, seen on the right leg (inner side), where SP-1 had been stimulated, and on the left arm (back side), where LI-1 had been stimulated. For the sole of the foot, no distance calculation was possible, as neither SP nor LR Meridians traverse this part of the body. On the inner side of the right leg (SP-1), agreement with the sensation mean course was better for the SP Meridian (*d* = 0.83 cm) than for the LR Meridian (*d* = 1.07 cm). However, this clear connection between stimulated point and associated meridian was not always found. For example, sensations on the right arm that should be related to stimulation of the point SI-1, showed good agreement with, both, the SI Meridian (*d* = 1.30 cm) and the HT Meridian (*d* = 1.39 cm). A general observation was the low variance of the distance: Maximum distances of sensation mean courses and meridians were as low as 1.28 cm (for SP-1 and the SP meridian) and 1.37 cm (for BL-67 and the BL meridian), meaning that the sensation never deviated more than this value from the meridian. Minimum distances showed points, where sensation mean courses virtually intersected with meridians (0.02 cm for LI-1 and the LI meridian, 0.06 cm for SI-1 and the HT meridian, and 0.38 cm for SI-1 and the SI meridian). Interestingly, due to our calculation method, such small values also imply a negligible variability in meridian courses. These points, where all lines come very close to each other, were all located on the distal part of the extremities (see Figure 3).

## 4. Discussion

In this paper, we have demonstrated the general feasibility of two experimental concepts: firstly, we have shown that propagated sensations along channels (PSC) can be studied under sensory deprivation in a single-blinded design, when using laser acupuncture. Secondly, we applied for the first time a geographic information system (GIS) to the study of PSC phenomena. 

We used sensory deprivation in an isolation tank to reduce environmental stimuli (visual, auditory, tactile, and temperature), to allow subjects to fully concentrate on sensations occurring during laser acupuncture stimulation. Judging from the ratio of occurrence of PSC (82.4%) in our experiment, we can say that this strategy seems to be a successful one, as other studies have reported much lower ratios: the larges cohort that has so far been investigated, comprised incredible 63,228 individuals in more than 20 districts in China in the late 1970s [24]. The authors reached the conclusion that PSC occurs in 20.7% of subjects, although these studies used needles instead of an acupuncture laser. Thus, the use of an isolation tank to study PSC in detail can be recommended despite the increased effort of such an endeavor.

Using laser instead of conventional needle acupuncture produced very similar sensations to those usually reported in studies on the acupuncture sensation *deqi*. *Tingling, radiating, spreading, hot (warm), dull, *and* throbbing* are descriptors that have often been reported in this context [1, 3, 25]. However, also sham laser acupuncture has previously been reported to induce such sensations [26, 27]. This reminds us of our own observations of false-positive results, when subjects were asked, which points they believed had been stimulated. Only one of the single points (LI-1) showed a significant correlation of stimulation and sensation. Even more strangely, none of the subjects estimated the stimulation order correctly. Contemplating these results in conjunction with our rather small sample size, we must not neglect the possibility that the actual laser stimulation (i.e., laser on or off) may not be of central importance for the elicitation of *deqi* and PSC. In other words, the phenomenon of PSC may be unrelated to actual laser stimulation taking place. This should be tested in further experiments.

Within the GIS framework, we have introduced a new approach to calculate mean courses of line-like sensations, which now allows group analysis of spatial PSC patterns. This method can be used to compare PSC lines (i.e., sensation mean courses) to any other data, whose spatial pattern can be mapped on a body scheme similar to that used in our study (Supplementary Figure 1), which constitutes an important step forward in the study of the still unknown physiological basis of PSC and its relation to other acupuncture effects. In our study, PSC lines were compared to classical meridians, as this connection has been reported numerous times before [28]. Despite the purely descriptive character of our analysis, we believe that an average distance between sensation mean courses and meridians of around 1 cm, as observed in our study, clearly points towards a close connection of the two entities. We also sought to include possible variability of meridian courses between different literature sources [29] and, thus, developed an approach to incorporate this variability in the distance measurement by means of Euclidean distance fields. An interesting side observation was that variability seemed to be larger for some meridians than for others. Meridians with a rather smooth course, like HT, LI, KI, and SP showed less variability than those with sharp edges, like SI and BL. Although not the focus of this study, GIS may in general provide a means for further investigation of meridian variability. This should be of importance for the study of the underlying mechanism of PSC as well as acupuncture in general. We speculate that the paradigmatic concept of meridians as small-diameter, line-like vessels with clear anatomical courses cannot withstand closer investigation.

Although this study was more like a proof-of-concept, some limitations need to be addressed:

Firstly, the small sample size precludes a detailed interpretation especially of the results concerning the connection of stimulated and sensed points. Furthermore, a design where only two of the four points had been stimulated, would have probably made the results clearer in the sense that it would have been easier to achieve significant results in Fisher's exact test. One could also argue, that we have not tested, how well the participants of the study were able to remember the sensations after each single laser stimulation. So they may given unreliable answers after receiving three stimulations in a row.

From the GIS-methodological point of view, the use of body schemes as a base map within the GIS is not quite correct, as the complex 3-dimensional shape of the human body is transferred into 2D by a simple orthogonal projection. In cartography, the shape of the Earth is described by 3D bodies such as spheres (for small scales), ellipsoids (usually locally optimized), or the geophysical Geoid model (for highest precision) and transferred to the plane by a map projection. The wide choice of possible projections allows preserving selected metric properties such as areas or local angles. Both 3D models for representing the shape of the Earth and map projection methods are integrated into GIS software, but there is, so far, no “standard human body” model implemented in these systems. In our case, the body maps do not precisely preserve areas or distances, resulting in distortions mainly around the edges of the body maps. Therefore, small differences in calculated distances, for example between meridians running along the middle of the body trunk and along the sides of the body must be interpreted with caution.

Furthermore, our choice of meridians for distance calculation might be criticized, as we did not restrict ourselves to the meridian connected to the stimulated point (e.g., the SP meridian for the point SP-1), but also included other meridians with a known connection to the stimulated finger or toe. This was done, after first results showed that many subjects experienced PSC following the course of the HT meridian, despite SI-1 being the stimulation locus. Such “jumping” of PSC from one meridian to an adjacent one has been reported from the very beginning of PSC research in China [24] and we believe that despite the obvious connection between PSC and meridians, the latter may be the result of some oversystematization of early PSC observations during the last two millennia. 

For the future, it would be desirable to make the transition from descriptive to predictive PSC analyses using GIS. This could be accomplished by including null data for comparison. Such null data may either be generated by some model or by changing the position and shape of existing objects with Monte-Carlo-like methods. Once this is accomplished, *P* values could be calculated expressing the likelihood of findings, like those presented here, thus paving the way to finally understand the physiological underpinnings of *deqi*, PSC, and acupuncture in general.

## Supplementary Material

Supplementary Figure 1: The body scheme, that was used by the subjects to sketch their sensations during laser acupuncture. The arrow heads mark the points of stimulation.
Supplementary Figure 2: Methodological approach to measure distances from a cohort of meridians. The left picture shows the variability of different sources concerning the course of the kidney (KI) meridian. The right picture shows the Euclidean distance field that was then intersected with the mean center lines (see Figure 3+4 and Table 3 in the main manuscript).
Supplementary Table 1: Numeric results of the GIS analysis of acupuncture sensation patterns. Sensation intensities are also shown. The index counts disjunct sensation patterns of the same type for each subject. For line-like and 2-dimensional sensations, "total length" and "total area" indicate the sum of all disjunct patterns.

## Figures and Tables

**Table 1:** The most frequently used descriptors chosen by the subjects to describe their sensations during laser acupuncture. Only descriptors that were chosen by at least five subjects are shown.

Subjects	Descriptors										
	Tingling	Radiating	Spreading	Hot	Dull	Pulsing	Throbbing	Pricking	Stinging	Tender	Pinching
1	X	X									
2	X		X		X					X	
3		X		X			X				
4	X	X	X	X		X	X	X			X
5	X	X	X	X	X	X		X	X		
6	X	X	X		X			X			
7	X	X	X		X	X	X	X	X		
8	X										X
9					X					X	
10				X							
11				X						X	
12	X					X	X			X	
13						X	X	X	X		X
14	X	X	X	X	X	X	X				
15		X	X	X	X					X	
16	X	X	X	X			X	X	X		X
17	X	X	X			X		X	X		X

Sum	11	10	9	8	7	7	7	7	5	5	5

**Table 2:** Comparison of subjects' perceived and actually exerted stimulation. Fisher's exact test was calculated independently for each of the four stimulated points as well as for the overall effect.

Subjects	Left little toe (BL-67)				Right big toe (SP-1)				Left index finger (LI-1)				Right little finger (SI-1)				All points			
	Hits	Misses	Correct rejects	False positives	Hits	Misses	Correct rejects	False positives	Hits	Misses	Correct rejects	False positives	Hits	Misses	Correct rejects	False positives	Hits	Misses	Correct rejects	False positives
1			X			X			X					X			1	2	1	0
2	X							X	X				X				3	0	0	1
3	X						X		X				X				3	0	1	0
4				X	X				X					X			2	1	0	1
5	X				X						X		X				3	0	1	0
6	X				X						X		X				3	0	1	0
7	X				X				X							X	3	0	0	1
8				X	X					X			X				2	1	0	1
9	X					X			X						X		2	1	1	0
10	X					X			X						X		2	1	1	0
11		X						X	X					X			1	2	0	1
12	X					X					X						1	1	1	0
13	X						X		X					X			2	1	1	0
14	X				X							X	X				3	0	0	1
15		X						X		X			X				1	2	0	1
16	X				X						X		X				3	0	1	0
17	X				X				X						X		3	0	1	0

Sum	12	2	1	2	8	4	2	3	10	2	4	1	8	4	3	1	38	12	10	7

Fisher's exact test	*P* = 0.46				*P* = 1.00				*P* = **0.03**				*P* = 0.26				*P* = **0.02**			

**Table 3:** Distances of sensation mean courses to meridians at the same limb.

	Stimulated point	Front/back	Length of sensation mean course/cm	Meridian	Distance to meridian/cm		
					Min	Mean	Max
Front	Right big toe (SP-1)	f	25.00	SP	0.46	0.83	1.28
	Right big toe (SP-1)	f	25.00	LR	0.61	1.07	2.03
	Right little finger (SI-1)	f	27.07	HT	0.06	1.39	2.53
	Left little toe (BL-67)	f	11.71	KI	4.05	6.27	10.08

Back	Left little toe (BL-67)	b	20.28 (lower line)	BL	1.06	3.38	4.53
	Left little toe (BL-67)	b	16.18 (upper line)	BL	1.22	1.30	1.37
	Left index finger (LI-1)	b	30.35	LI	0.02	0.83	2.53
	Right little finger (SI-1)	b	27.88	SI	0.38	1.30	1.82
