# Supplementary material for: Investigation of Acupuncture Sensation Patterns under Sensory Deprivation Using a Geographic Information System
Source: Evid Based Complement Alternat Med. 2012 Nov 21;2012:591304. doi: 10.1155/2012/591304 (PMC3518766; doi:10.1155/2012/591304)
Supplement: Supplementary file 1 — Supplementary Figure 1: The body scheme, that was used by the subjects to sketch their sensations during laser acupuncture. The arrow heads mark the points of stimulation. Supplementary Figure 2: Methodological approach to measure distances from a cohort of meridians. The left picture shows the variability of different sources concerning the course of the kidney (KI) meridian. The right picture shows the Euclidean distance field that was then intersected with the mean center lines (see Figure 3+4 and Table 3 in the main manuscript). Supplementary Table 1: Numeric results of the GIS analysis of acupuncture sensation patterns. Sensation intensities are also shown. The index counts disjunct sensation patterns of the same type for each subject. For line-like and 2-dimensional sensations, "total length" and "total area" indicate the sum of all disjunct patterns. [file 591304.f1.pdf]

Supplementary Material

Investigation of acupuncture sensation patterns under sensory deprivation using a geographic information system

Florian Beissner,[1] Irene Marzolff [2]

[1] Pain & Autonomics – Integrative Research (PAIR), University Clinic of Psychiatry and Psychotherapy, Friedrich Schiller University Jena, Germany

[2] Institute for Physical Geography, Goethe University, Frankfurt am Main, Germany

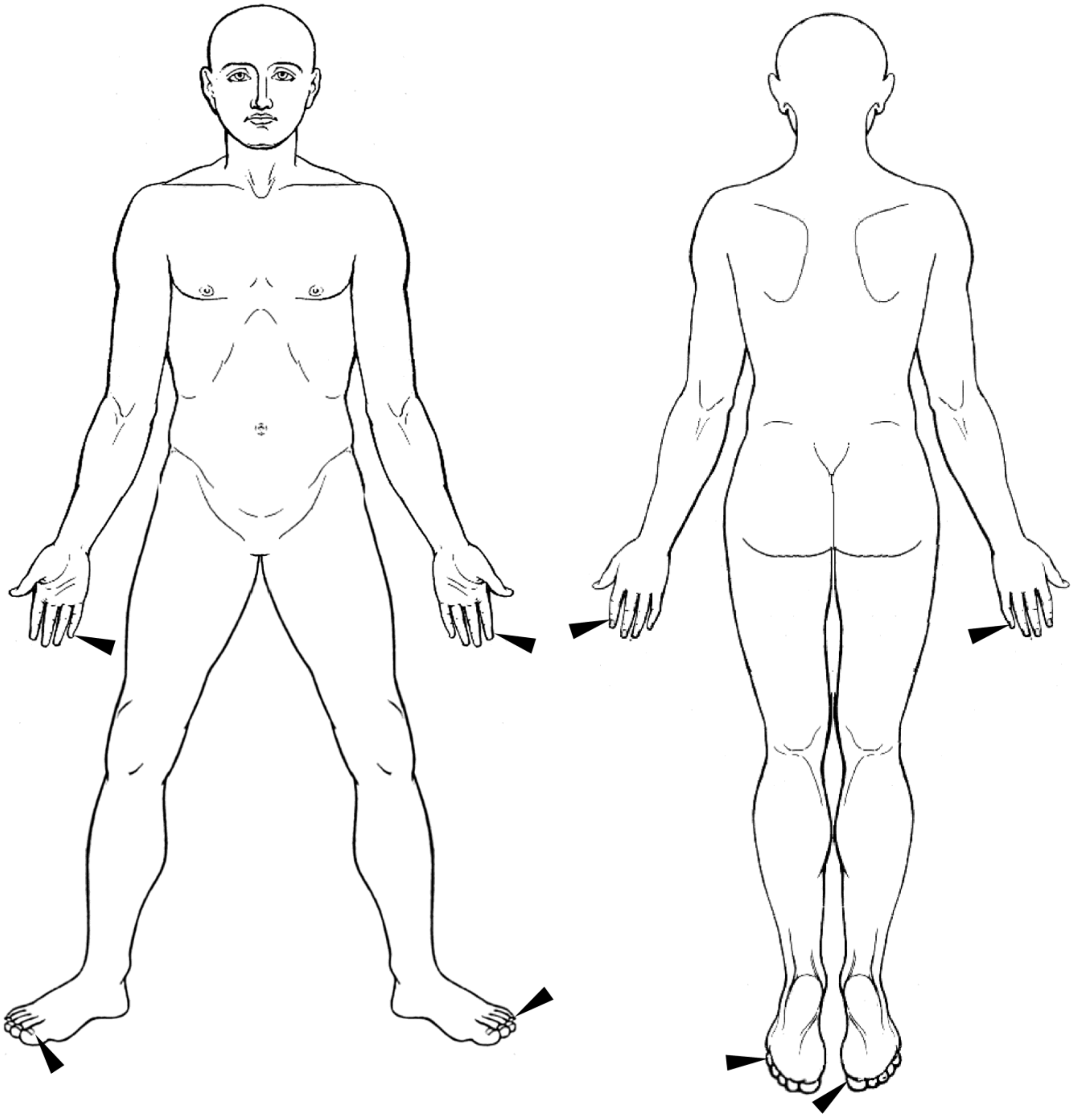

Supplementary Figure 1: The body scheme, that was used by the subjects to sketch their sensations during laser acupuncture. The arrow heads mark the points of stimulation.

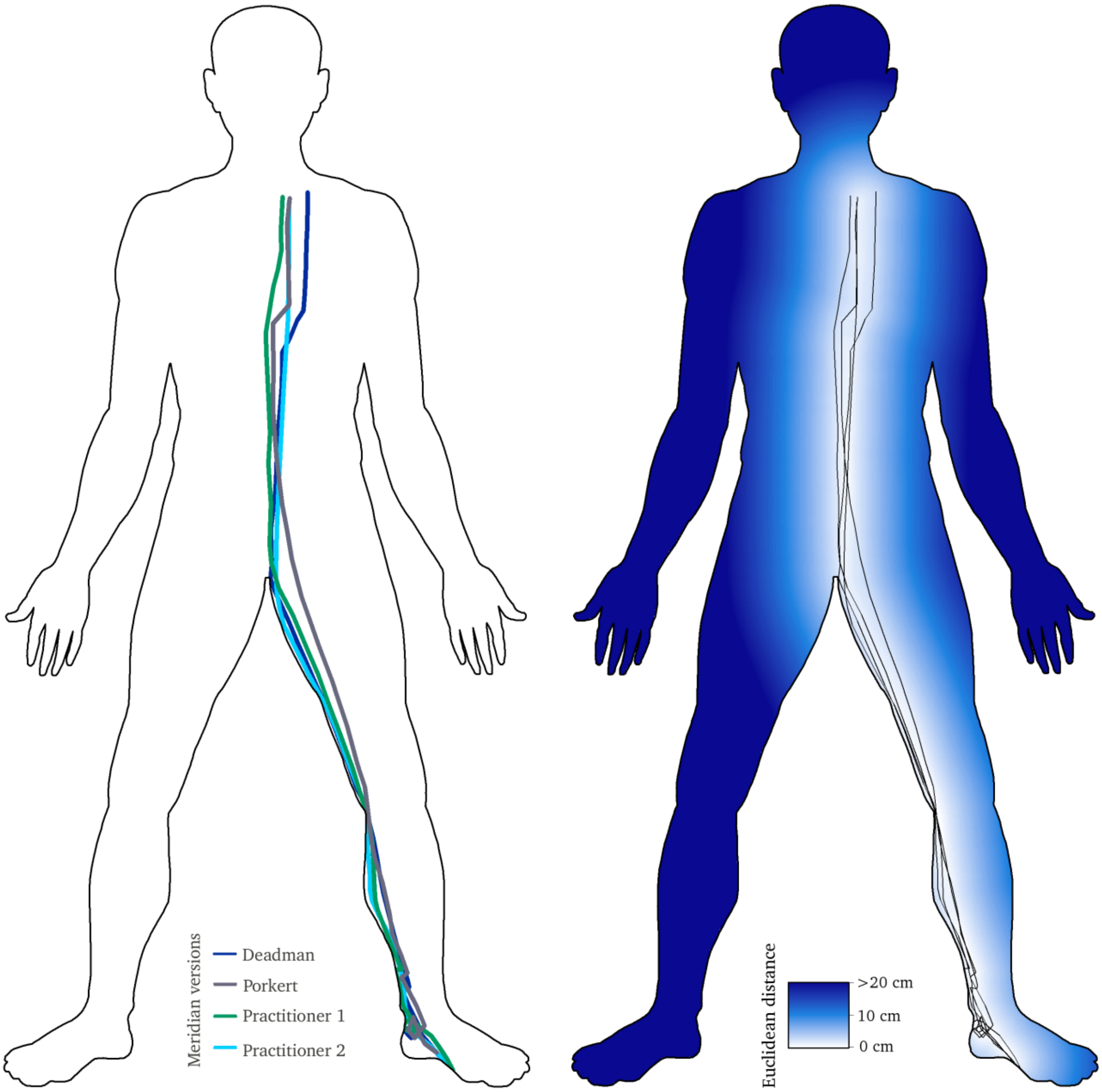

Supplementary Figure 2: Methodological approach to measure distances from a cohort of meridians. The left picture shows the variability of different sources concerning the course of the kidney (KI) meridian. The right picture shows the Euclidean distance field that was then intersected with the mean center lines (see Figure 3+4 and Table 3).

	Sensation intensity	point-like sensations			line-like sensations				2-dimensional sensations			
**Subject**	**VAS**	**front / back**	**Index**	**Radius/ cm**	**front / back**	**Index**	**Length/ cm**	**total Length / cm**	**front / back**	**Index**	**Area/ cm²**	**total Area/ cm²**
**1**	**6**							**0.00**				**0.00**
**2**	**21**	b	I	1.60	b	I	3.14	**37.21**	b	I	8.28	**166.31**
		b	II	0.50	b	II	3.24		b	II	9.40	
		f	I	0.50	b	III	3.19		b	III	64.82	
					f	I	10.07		b	IV	8.39	
					f	II	9.96		b	V	32.91	
					f	III	2.57		b	VI	30.65	
					f	IV	2.47		f	I	11.86	
					f	V	2.58					
**3**	**39**				b	I	49.88	**49.88**	b	I	31.56	**487.61**
									b	II	25.57	
									b	III	41.91	
									b	IV	47.07	
									b	V	27.58	
									f	I	156.88	
									f	II	157.03	
**4**	**31**	b	I	1.00	b	I	7.98	**7.98**	b	I	11.10	**206.01**
		b	II	1.40					b	II	11.65	
		b	III	1.00					b	III	10.31	
		b	IV	1.60					b	IV	8.74	
		f	I	1.00					b	V	7.16	
		f	II	1.00					f	I	39.67	
									f	II	40.75	
									f	III	76.62	
**5**	**42**	b	I	1.20	b	I	33.85	**138.13**	b	I	24.09	**473.60**
		b	II	1.00	f	I	19.64		b	II	85.77	
		b	III	1.70	f	II	15.24		f	I	363.74	
		f	I	1.00	f	III	9.00					
					f	IV	24.66					
					f	IX	5.37					
					f	V	8.86					
					f	VI	8.57					
					f	VII	8.61					
					f	VIII	4.36					
**6**	**37**	b	I	0.70	f	I	19.53	**68.24**	b	I	259.18	**340.64**
		b	II	2.80	f	II	14.48		b	II	81.46	
		f	I	0.70	f	III	6.59					
					f	IV	17.93					
					f	V	3.68					
					f	VI	3.54					
					f	VII	2.48					
**7**	**72**	b	I	1.00	f	I	40.43	**65.28**	b	I	14.75	**148.87**
					f	II	24.86		b	II	18.30	
									b	III	26.17	
									f	I	38.08	
									f	II	7.86	
									f	III	26.26	
									f	IV	17.44	
**8**	**5**	b	I	1.00	b	I	6.67	**12.97**	b	I	19.58	**41.16**
		b	II	2.50	f	I	6.30		f	I	13.91	
		f	I	0.90					f	II	7.67	
**9**	**6**	f	I	1.80				**0.00**				**0.00**

**10**	**10**				b	I	15.54	**15.54**	b	I	11.27	**717.39**
									b	II	399.50	
									b	III	7.34	
									f	I	15.06	
									f	II	284.22	
**11**	**14**	b	I	0.60	f	I	39.77	**39.77**	b	I	47.41	**47.41**
**12**	**15**	b	I	0.90				**0.00**				**0.00**
**13**	**18**	b	I	0.50				**0.00**				**0.00**
		b	II	1.00								
		f	I	0.90								
**14**	**24**	b	I	0.70	b	I	8.51	**105.15**	b	I	24.34	**24.34**
					b	II	27.20					
					f	I	14.73					
					f	II	25.99					
					f	III	28.73					
**15**	**18**							**0.00**	b	I	128.35	**409.74**
									f	I	281.38	
**16**	**23**	b	I	4.50	b	I	17.22	**61.79**	b	I	8.63	**8.63**
		b	II	0.70	b	II	6.64					
		b	III	1.60	b	III	8.47					
		b	IV	5.00	b	IV	11.27					
		b	V	4.50	b	V	9.76					
		b	VI	4.50	b	VI	8.41					
		b	VII	4.50								
		f	I	4.00								
**17**	**69**	b	I	1.20	b	I	4.97	**47.22**	b	I	16.48	**361.24**
		b	II	2.00	b	II	37.75		b	II	55.08	
		b	III	0.50	b	III	4.50		b	III	193.61	
									f	I	96.07	
**mean**	**26.47**			**1.67**			**13.81**	**40.50**			**67.31**	**201.94**

Supplementary Table 1: Numeric results of the GIS analysis of acupuncture sensation patterns. Sensation intensities are also shown. The index counts disjunct sensation patterns of the same type for each subject. For line-like and 2-dimensional sensations, "total length" and "total area" indicate the sum of all disjunct patterns.
